# Transgenerational Glucose Intolerance of Tumor Necrosis Factor with Epigenetic Alteration in Rat Perirenal Adipose Tissue Induced by Intrauterine Hyperglycemia

**DOI:** 10.1155/2016/4952801

**Published:** 2016-01-05

**Authors:** Rina Su, Jie Yan, Huixia Yang

**Affiliations:** Department of Obstetrics and Gynecology, Peking University First Hospital, No. 8, Xishiku Street, Xicheng District, Beijing 100034, China

## Abstract

Changes in DNA methylation may play a role in the genetic mechanism underlying glucose intolerance in the offspring of mothers with diabetes. Here, we established a rat model of moderate intrauterine hyperglycemia induced by streptozotocin to detect glucose and lipid metabolism of first-generation (F1) and second-generation (F2) offspring. Moderate intrauterine hyperglycemia induced high body weight in F1 and F2 offspring of diabetic mothers. F1 offspring had impaired glucose tolerance and abnormal insulin level. Additionally, F1 and F2 offspring that were exposed to intrauterine hyperglycemia had impaired insulin secretion from the islets. The tumor necrosis factor (*Tnf*) gene was upregulated in perirenal adipose tissue from F1 offspring and relatively increased in F2 offspring. Both F1 and F2 offspring showed similar hypomethylation level at the −1952 site of* Tnf*. We confirmed that DNA methylation occurs in offspring exposed to intrauterine hyperglycemia and that the DNA methylation is intergenerational and inherited.

## 1. Introduction

Gestational diabetes mellitus (GDM) is characterized by intrauterine hyperglycemia and has been reported to affect 17.5% of pregnant women in China [1]. GDM is associated with health issues in offspring. The offspring of mothers with GDM have been shown to have higher birth weights and are reportedly prone to obesity, hypertension, and dyslipidemia compared to offspring of mothers without diabetes [2, 3]. It has been suggested that, apart from genetic influences, the intrauterine environment may also influence the phenotype of the offspring.

“Programming” refers to the process by which a stimulus at a critical window of development has long-term effects. Numerous studies have investigated the effects of adverse intrauterine environment and found that it is correlated with poor fetal growth and increased risk of type 2 diabetes and obesity in adulthood [4]. Some studies have shown that obesity and type 2 diabetes are a global metabolic disorder and systemic inflammation, and the intersection of these phenomena is especially evident in visceral adipose tissue [5, 6]. Nevertheless, the potential mechanism of glucose intolerance induced by intrauterine hyperglycemia remains to be fully understood, and it is yet unknown whether inflammation is related to metabolic disturbances in offspring exposed to intrauterine hyperglycemia.

It has been suggested that epigenetic mechanisms may be the link between environmental and nutritional factors and regulation of gene expression. DNA methylation is one of the major epigenetic modifications. We hypothesized that changes in DNA methylation could participate in the expression of genes related to glucose intolerance in offspring. Furthermore, DNA methylation might also determine the transgenerational disease transmission.

In this study, we established a rat model of streptozotocin- (STZ-) induced moderate intrauterine hyperglycemia. F1 female rats were fed either normal diet or high-fat diets after weaning. The F1 adult female rats obtained from the control and diabetic rats were mated with control male rats, and the phenotypes of F2 female offspring were characterized to study transgenerational influences. Furthermore, we analyzed the methylation status in perirenal adipose tissue to investigate whether intrauterine hyperglycemia affected gene expression of inflammatory cytokines by regulating epigenetic modification.

## 2. Materials and Methods

### 2.1. Animals and Tissue Isolation

**Figure 1:**
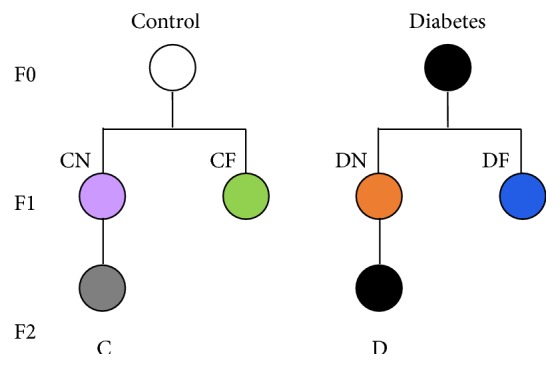
GDM rat model with moderate hyperglycemia induced by streptozotocin. Study design: female offspring were fed either normal or high-fat diets after weaning: control groups were fed normal diet (F1-CN) or high-fat diet (F1-CF) and rats in the diabetic group were fed normal diet (F1-DN) or high-fat diet (F1-DF). Adult females of the F1-CN and F1-DN groups were mated with control males to obtain F2 female offspring (F2-C and F2-D). Purple denotes the F1-CN group; green, F1-DN; orange, F1-CF; blue, F1-DF; and grey, F2-C group; and the plain circle denotes the F2-D group.

Twenty-one Wistar rats were used in this study. All the rats were fed in the specific-pathogen-free (SPF) grade animal test room with stable room temperature and humidity. The rats were housed in a 12 : 12 light : dark cycle. All animal protocols were reviewed and approved by the Institutional Animal Care and Use Committee of Peking University First Hospital (J201010). At 12 weeks, female Wistar rats (Vital River Laboratory Animal Technology Co., Ltd., Beijing, China) were mated with male rats. Onset of pregnancy was determined by the presence of a vaginal plug after overnight mating (designated as day 0 [D0] of pregnancy). After the rats were made to fast for a 12 h period, the female rats were randomly divided into two groups: intrauterine hyperglycemia group (F0-D), in which fourteen rats were injected with a single intraperitoneal injection of 25 mg/kg STZ (Sigma) in citrate buffer (pH 4.4) on D0.5, and control group (F0-C), in which the seven pregnant rats received an equal volume of citrate buffer on D0.5. On D4 of pregnancy and every three days thereafter, the blood glucose concentration was measured via the tail vein to check for diabetes. The pregnant rats were allowed to deliver spontaneously. Female offspring were studied and fed either a normal diet (12% kcal fat, 24% kcal protein, and 64% kcal carbohydrates; Beijing KeAoXieLi Feeds Co., Ltd., Beijing, China) or a high-fat diet (45% kcal fat, 20% kcal protein, and 45% kcal carbohydrates; Beijing KeAoXieLi Feeds Co., Ltd., Beijing, China) after weaning: the control group rats were fed a normal diet (F1-CN, *n* = 9) or high-fat diet (F1-CF, *n* = 9), and the diabetic group rats were fed a normal diet (F1-DN, *n* = 9) or high-fat diet (F1-DF, *n* = 9). The F1-CN and F1-DN female adult rats were then mated with control male rats. The phenotypes of F2 female offspring were characterized. F2 female offspring were divided into F2-C and F2-D groups (Figure 1). The F1 and F2 offspring were sacrificed at 28 weeks. Blood from the abdominal aorta was obtained and centrifuged at 3,000 ×g for 10 min to separate the serum and stored at −80°C until analysis. The heart, liver, pancreas, kidney, and fat pads (including mesenteric, perirenal, and ovarian fat) were carefully dissected and weighed. Perirenal adipose tissues were dissected from visible blood vessels and immediately frozen in liquid nitrogen, and the perirenal adipose tissue samples were stored at −80°C before further processing.

### 2.2. Oral Glucose Tolerance Test

F1 rats from all the four F1 groups (F1-CN, F1-DN, F1-CF, and F1-DF) were fasted overnight and given 2 g/kg glucose by gavage for the oral glucose tolerance test (OGTT) conducted at 16, 20, and 24 weeks. Blood samples were collected from the tail vein before (*t* = 0) and at 30, 60, and 120 min of glucose administration.

### 2.3. Biochemical Test and Analysis

The fasting insulin (FINS) levels were measured in the F1-CN, F1-DN, F2-C, and F2-D groups by enzyme-linked immunosorbent assay (ELISA) at 28 weeks (rat insulin ELISA kit, Cayman Chemical, 589501, USA), according to the manufacturer's instructions. The total triglyceride (TG) and high-density lipoprotein (HDL) levels in the F1-CN, F1-DN, F2-C, and F2-D groups were detected in a fully automatic biochemical analyzer at 28 weeks. The reagents used in the assays were provided by Beijing BHKT Clinical Reagent Co., Ltd., China.

### 2.4. Histology and Morphometric Analysis

Morphological evaluation was carried out as follows: briefly, pancreases were dissected rapidly, cleaned of connective tissue, weighed, and fixed in 4% paraformaldehyde solution for 48 h. The pancreases were then embedded in wax and serially sliced to obtain 5 *μ*m wide histological sections. The sections were stained with hematoxylin and eosin and then the morphological features were analyzed under a microscope. The areas and diameters of pancreatic islets were measured using Image-Pro Plus 6.0 (Media Cybernetics). Ten different fields were analyzed per rat, and six rats in each of the F1-CN, F1-DN, F2-C, and F2-D groups were analyzed.

### 2.5. Immunohistochemistry of Pancreatic Islets

Immunolocalization was performed for anti-insulin reaction on 5 *μ*m thick sections, and the sections were subsequently stained with streptavidin biotin peroxidase. Paraffin sections were deparaffinized in xylene and rehydrated in descending grades of alcohol followed by heat-induced epitope retrieval. Endogenous peroxidase and nonspecific antibody binding sites were suppressed by treating the sections with 0.3% hydrogen peroxide for 10 min at room temperature. The sections were then washed in phosphate-buffered saline and incubated for 1 h with primary antibodies for rat insulin (ZSGB-BIO, Beijing, China). The sections were washed again with phosphate-buffered saline and incubated with biotinylated secondary antibody (PV6000, ZSGB-BIO, Beijing, China) for 40 min. Finally, the nuclei were stained using hematoxylin and eosin and mounted in 3,3′-diaminobenzidine (DAB). Antibody binding was evaluated by high-power light microscopy (cellSens, Olympus DP72, Japan). The pancreatic islet area and fluorescence intensity were measured using Image-Pro Plus 6.0 (Media Cybernetics). Ten different fields were analyzed per rat, and six rats per group were analyzed.

### 2.6. MeDIP Array

A purified genomic DNA pool was prepared from three perirenal adipose tissue samples from the F1-CN and F1-DN groups and digested overnight with* Mse*I restriction enzyme (NEB, R0525S). Denatured DNA was immunoprecipitated using monoclonal antibodies against 5-methyl cytidine (Abcam, Ab10805). Immunoprecipitated DNA was recovered with proteinase K digestion followed by column-based purification, amplified, and hybridized on a Rat DNA Methylation 3 × 720 K CpG Island Plus RefSeq Promoter Array according to the protocol of Roche NimbleGen. Immunoprecipitated (IP) and input DNA samples were labeled with Cy5 and Cy3, respectively. Hybridization was performed using a NimbleGen Hybridization Kit and NimbleGen Hybridization System 12. Scanning was done using high-resolution NimbleGen MS 200 Microarray Scanner. MeDIP array data were analyzed using NimbleScan software, and the array results could be visualized using SignalMap software. Microarray data reported in the paper were deposited to the Gene Expression Omnibus (GEO) at the National Center for Biotechnology Information (NCBI) under the series accession number GSE65779.

### 2.7. DNA Methylation ChIP Analysis

MeDIP samples from the F1-CN and F1-DN groups were hybridized to Rat DNA Methylation 3 × 720 K CpG Island Plus RefSeq Promoter Array representing 15,287 putative promoters (−3.88 kb to +0.97 kb to the transcription start site (TSS)) and 15,790 CpG islands. Chromatin immunoprecipitation (ChIP) analysis was carried out by CapitalBio Corporation (Beijing, China). We identified 923 and 2150 promoters as significantly hypermethylated and significantly hypomethylated, respectively. The biological significance of the microarray data was analyzed by the Molecular Annotation System (CB-MAS, CapitalBio Corporation, Beijing, China, http://bioinfo.capitalbio.com/mas3/).

### 2.8. DNA Methylation Analysis Using Bisulfite Sequencing

Genomic DNA of three perirenal adipose tissue samples from the F1-CN, F1-DN, F2-C, and F2-D groups was treated and purified with the EpiTect bisulfite kit (Qiagen), according to the manufacturer's instructions. The methylation status was evaluated by cloning and sequencing the bisulfite-treated DNA. The following primers were used to amplify region −2012 to −1829 of the* Tnf* promoter: sense 5′-GGT TTT TTT TGG AGA AAG TTG TTT-3′, antisense 5′-AAA AAC ACA ACC CCC TAA TAC ATT A-3′. The following primers were used to amplify region −220 to +26 of the* Tnf* promoter: sense 5′-TTT TGA TGT TTG GGT GTT TTT AAT T-3′, antisense 5′-TTC TCC CTC CTA ACT AAT CCC TTA A-3′. The purified PCR products were cloned using a pEASY-T1 Simple Cloning Vector system (TransGen Biotech, Beijing, China), and ten individual clones in each sample were sequenced. A total of three samples were evaluated in each group. The sequence obtained by cloning was analyzed at Beijing General GeneTest Co., Ltd. (Beijing, China).

### 2.9. Nucleic Acid Purification and Real-Time PCR

For RNA extraction, perirenal adipose tissues from the F1-CN, F1-DN, F2-C, and F2-D groups were homogenized in 1 mL of TRIzol reagent and RNA was purified according to the manufacturer's recommendations. The RNA was used as a template for synthesis of the same amount of cDNA (2 *μ*g) using First Strand Synthesis Kit (Applied Biosystems, USA). cDNA quantity was measured using real-time PCR with the ABI PRISM 7500 sequence detection system and fluorescence-based SYBR Green technology. The PCR reaction mixture consisted of 2 *μ*g of diluted cDNA sample, 2x SYBR Green PCR Master Mix (Molecular Probes, USA), primers optimized for each target gene, and nuclease-free water to achieve a final volume of 20 *μ*L. All the samples were analyzed in duplicate. Primers were designed using Primer Express 3.0 software. The following primers were used:* Rplp0*, sense 5′-GGC GAC CTG GAA GTC CAA-3′ and antisense 5′-TCT GCT CCC ACA ATG AAG CA-3′;* Tnf*, sense 5′-TGA TCG GTC CCA ACA AGG A-3′ and antisense 5′-GGG CCA TGG AAC TGA TGA GA-3′;* IL-1β*, sense 5′-CCCAAGCACCTTCTTTTCCTT-3′ and antisense 5′-CGTCATCATCCCACGAGTCA-3′;* IL-6*, sense 5′-ACAGAGGATACCACCCACAACAG-3′ and antisense 5′-TCAGAATTGCCATTGCACAAC-3′;* IL-10*, sense 5′-CAGTCAGCCAGACCCACATG-3′ and antisense 5′-TGTTGTCCAGCTGGTCCTTCT-3′;* IFN-γ*, sense 5′-ATCGAATCGCACCTGATCACT-3′ and antisense 5′-GTGCTGGATCTGTGGGTTGTT-3′.

### 2.10. Western Blot Analysis

Perirenal adipose biopsies from the F1-CN, F1-DN, F2-C, and F2-D groups were homogenized in RIPA lysis buffer (KeyGen Biotech, KGP702, Nanjing, China) and 1 mM phenylmethylsulfonyl fluoride (PMSF; Amresco, M221, USA). The protein content was determined using a bicinchoninic acid (BCA) protein assay kit from KeyGen Biotech (Nanjing, China). Protein samples were placed at equal concentrations on polyacrylamide gel and separated using 12% sodium dodecyl sulfate-polyacrylamide gel electrophoresis (SDS-PAGE). Proteins were transferred to polyvinylidene difluoride (PVDF) membranes (Applygen, P2110, Beijing, China) and subjected to Western blot analysis. After incubation with primary antibodies (Cell Signaling Technology, 11948S, USA) overnight, the PVDF membranes were washed and incubated with horseradish peroxidase-conjugated secondary antibodies (ZSGB-BIO, Beijing, China) for 1 h at room temperature. The results were quantified by densitometry using AlphaEaseFC FluorChem SA software for Windows (Alpha Innotech Corporation, California, USA).

### 2.11. Statistics

One-way ANOVA was used to determine the comparisons among the four groups. Post hoc comparisons using Tukey's test were performed when a significant *F*-score was detected. Comparisons between two groups were performed using two-tailed unpaired Student's *t*-test. All the values are presented as mean ± SEM. Statistically significant differences were defined at *p* < 0.05.

## 3. Results

### 3.1. Moderate Intrauterine Hyperglycemia Induced High Body Weight in F1 Females

As previously described [7], we established a moderate intrauterine hyperglycemia rat model by injecting Wistar rats with a single intraperitoneal injection of streptozotocin (STZ). The average glucose level of diabetic mothers during pregnancy was 11.2–15.0 mmol/L. Further morphological studies of the pancreas samples confirmed that rats with GDM had smaller pancreatic islets than rats without GDM.

The birth weight was significantly higher in the F1-DN group than in the F1-CN group (Table 1). The body weight was also significantly higher in the F1-DN group than in the F1-CN group at 15 weeks (Table 1). Furthermore, intrauterine hyperglycemia was found to induce adipopexis in F1 offspring, and the perirenal adipose tissue, ovarian adipose tissue, and whole visceral adipose tissue weighed more in the F1-DN group than in the F1-CN group; however, the tissue weights did not significantly differ between the two groups (Table 2). The pancreas, liver, heart, and kidney weights also showed no significant differences between the F1-DN and F1-CN groups (Table 2).

The body weight was higher in the F1-CF group than in the F1-CN group at 15 weeks (Table 1). The high-fat diet did not further exacerbate the effect of intrauterine hyperglycemia exposure on body weight (F1-DF versus F1-DN, Table 1).

The weights of perirenal adipose tissue and whole visceral adipose tissue were higher in the F1-CF group than in the F1-CN group with borderline significance (*p* = 0.065 and *p* = 0.094, resp.). There were no significant differences in the weights of the pancreas, liver, heart, and kidney in each group (Table 2).

### 3.2. Moderate Intrauterine Hyperglycemia Induced Glucose Intolerance and Abnormal Insulin and Lipid Levels in F1 Females

**Figure 2:**
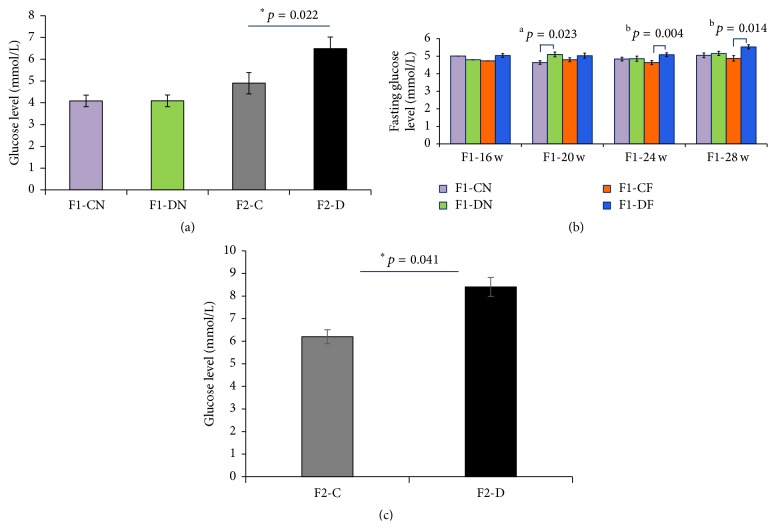
Glucose levels in F1 and F2 offspring. (a) The F2 offspring in the F2-D group have a higher glucose level at birth. (b) Rats in the F1-DN group have a higher fasting glucose level at 20 weeks, and rats in the F1-DF group have a higher fasting glucose level at 24 and 28 weeks than rats in the F1-CF group. (c) Rats in the F2-D group have a higher glucose level at 28 weeks. The results are means ± SEM, *N* = 8-9; ^a^ *p* < 0.05 versus F1-CN; ^b^ *p* < 0.05 versus F1-CF; ^*∗*^ *p* < 0.05 versus F2-C.

**Figure 3:**
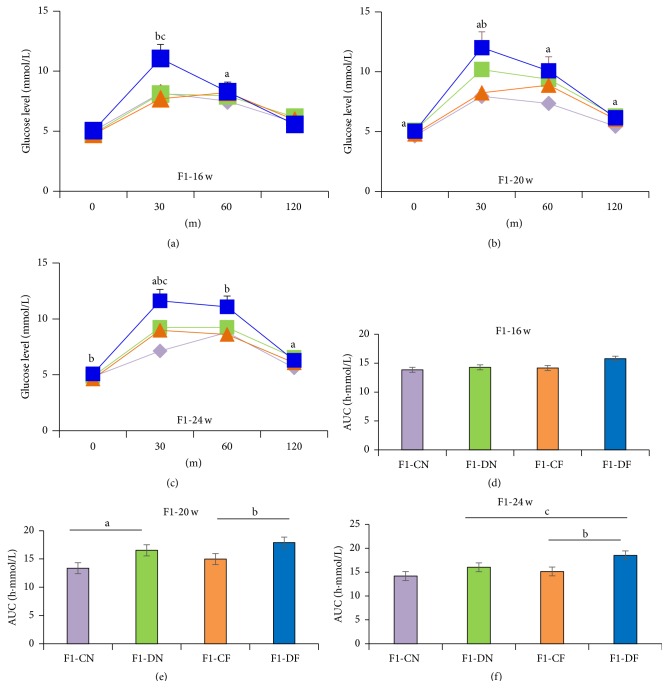
Glucose tolerance test. (a–c) Glucose tolerance test (GTT) was performed at 16, 20, and 24 weeks using 2 g glucose/kg body weight given by gavage. (d–f) AUC was performed at 16, 20, and 24 weeks. The results are presented as means ± SEM, *N* = 8-9; ^a^ *p* < 0.05 versus F1-CN; ^b^ *p* < 0.05 versus F1-CF; ^c^ *p* < 0.05 versus F1-DN.

At birth, there was no significant difference in the blood glucose level between the F1-CN and F1-DN groups (Figure 2(a)). The fasting glucose levels were monitored in the F1 rats at 16, 20, 24, and 28 weeks. The fasting glucose level was higher in the F1-DN group than in the F1-CN group at 20 weeks (Figure 2(b)). The fasting glucose level was also elevated in the F1-DF group compared to that in the F1-CF group at 24 and 28 weeks (Figure 2(b)). Results of the GTT revealed impaired glucose tolerance (IGT) in the F1-DN group at 20 weeks. In this group, the blood glucose level had significantly increased at 0, 30, 60, and 120 min after glucose load, and the AUC was significantly higher in the F1-DN group than in the F1-CN group (Figures 3(b) and 3(e)). The GTT revealed IGT at 30 and 120 min in the F1-DN group at 24 weeks, and there were no differences in the AUC between the F1-DN and F1-CN groups at 24 weeks (Figures 3(c) and 3(f)). The GTT revealed no difference in the glucose tolerance at 16 weeks (Figures 3(a) and 3(d)).

The high-fat diet also induced glucose intolerance. The blood glucose level significantly increased at 60 min after glucose load at 16 weeks, at 60 and 120 min at 20 weeks, and at 30 min at 24 weeks in the F1-CF group compared to the F1-CN group (Figures 3(a)–3(c)). The high-fat diet could exacerbate glucose intolerance of offspring exposed to intrauterine hyperglycemia, as evidenced by the blood glucose level, which was significantly increased at 30 min after glucose load at both 16 and 24 weeks in the F1-DF group compared to the F1-DN group, and the AUC was significantly increased at 24 weeks (Figures 3(a), 3(c), and 3(f)). Moreover, IGT was found in the F1-DF group compared to the F1-CF group, and the blood glucose level of the former group significantly increased at 30 min at 16 weeks; 30 min at 20 weeks; and 0, 30, and 60 min at 24 weeks. Furthermore, the AUC was significantly higher at 20 and 24 weeks (Figures 3(a), 3(b), 3(c), 3(e), and 3(f)).

**Figure 4:**
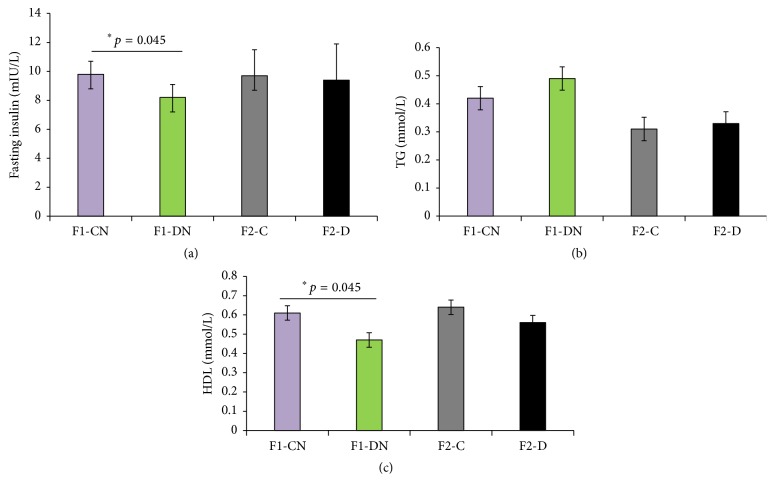
Biochemical analysis of F1 and F2 offspring at 28 weeks. (a) Level of fasting insulin in F1 and F2 offspring. (b) TG level in F1 and F2 offspring. (c) HDL level in F1 and F2 offspring. Results are presented as means ± SEM, *N* = 6 per group; ^*∗*^ *p* < 0.05 versus F1-CN.

The FINS, triglyceride, and HDL levels in the F1 rats were monitored at 28 weeks (Figures 4(a)–4(c)). The FINS and HDL levels were significantly decreased in the F1-DN group compared to the F1-CN group (Figures 4(a) and 4(c)).

### 3.3. Moderate Intrauterine Hyperglycemia Induced High Body Weight and Hyperglycemia in F2 Females

The birth weights of F2 females were similar between the diabetes and control groups, and body weight increased in the F2-D group compared to the controls at 23 and 28 weeks (Table 1). In addition, the blood glucose levels at birth and at 28 weeks were significantly increased in the F2-D group compared to those in the F2-C group (Figures 2(a) and 2(c)). Moreover, the weight of perirenal adipose tissue was significantly higher in the F2-D than that in the F2-C group, whereas the weights of the pancreas, liver, heart, and kidney did not significantly differ between the two groups (Table 2). In addition, the FINS, TG, and HDL levels were similar between the two F2 groups (Figures 4(a)–4(c)).

### 3.4. Moderate Intrauterine Hyperglycemia Induced Transgenerational Effects on Islet Structure and Impaired Insulin Secretion

**Figure 5:**
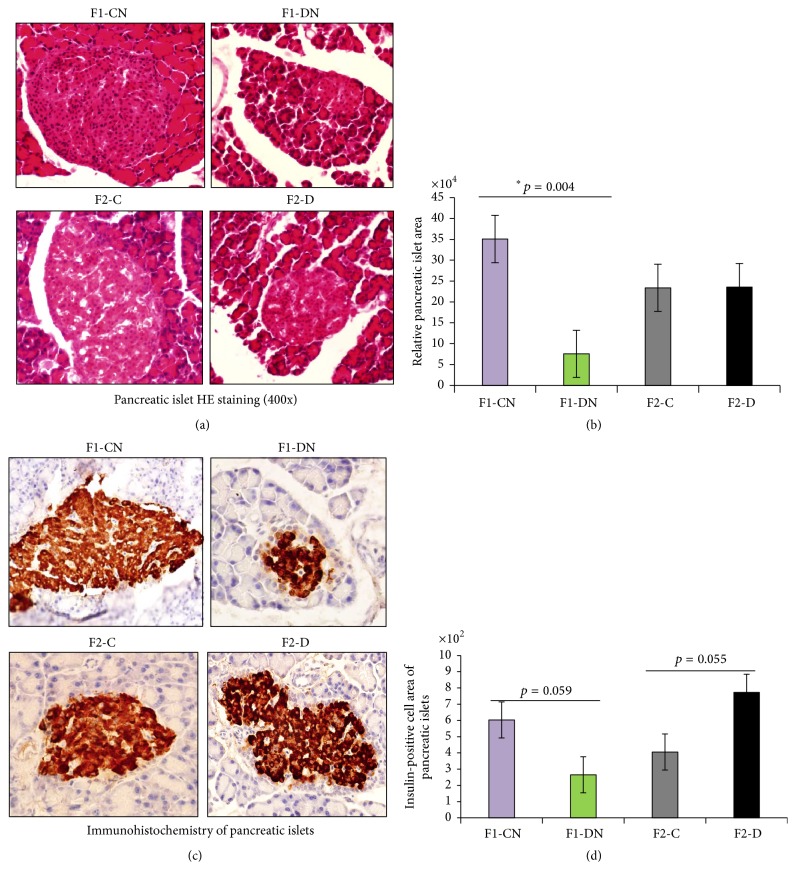
Histology and immunohistochemistry of pancreatic islets. (a) HE staining images (400x) of pancreatic islets obtained from F1 and F2 offspring. (b) The area and diameters of pancreatic islets were measured using Image-Pro Plus 6.0, Media Cybernetics. Ten different fields were analyzed for each rat, with a total of six rats per group. (c) Immunohistochemistry of pancreatic islets. (d) Insulin-positive cell area of pancreatic islets was measured using Image-Pro Plus 6.0. Ten different fields were analyzed for each rat; totally six rats in each group were analyzed. Results are presented as means ± SEM, *N* = 6 per group; ^*∗*^ *p* < 0.05 versus F1-CN.

A morphological study of the pancreas samples confirmed that the F1-DN group had obviously smaller pancreatic islets and atrophied *β*-cell mass compared to the F1-CN group (Figures 5(a) and 5(b)). Pancreatic islets in the F2-D group were similar to those in the control group (Figures 5(a) and 5(b)). To further define the potential secretory defects that may be related to the abnormal structure of islets in offspring exposed to intrauterine hyperglycemia, pancreatic islets were immunohistochemically analyzed. The level of insulin-positive cells in the islets was borderline significantly reduced in F1-DN group compared to F1-CN group (Figures 5(c) and 5(d)); interestingly, the level of insulin-positive cell area of islets was borderline significantly increased in F2-D group compared to that in F2-C group (Figures 5(c) and 5(d)).

### 3.5. Altered Genome-Wide Methylation Levels in F1 Female Offspring Exposed to Intrauterine Hyperglycemia

We used methylated DNA immunoprecipitation (MeDIP) combined with microarray technology to discover whether changes in DNA methylation are specific to intrauterine hyperglycemia exposure. We identified differentially methylated genes in the F1-DN group using Rat DNA Methylation 3 × 720 K CpG Island Plus RefSeq Promoter Array representing 15,287 putative promoters (–3.88 kb to +0.97 kb respective to the TSS) and 15,790 CpG islands. A total of 923 and 2150 promoters were significantly hypermethylated and hypomethylated, respectively, compared to the controls in perirenal adipose tissue.

**Figure 6:**
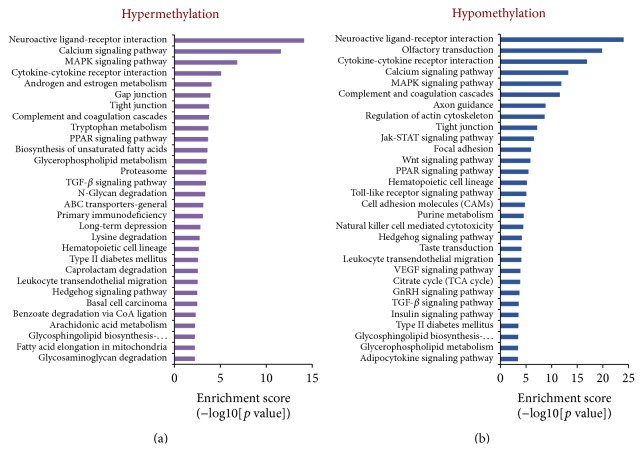
The pathways of DNA methylation changes in F1 offspring exposed to intrauterine hyperglycemia. Immunoprecipitated DNA was amplified and hybridized on Rat DNA Methylation 3 × 720 K CpG Island Plus RefSeq Promoter Array according to the Roche NimbleGen protocol. Microarray data was extracted and analyzed by the Molecular Annotation System (CB-MAS, CapitalBio Corporation, Beijing, China, http://bioinfo.capitalbio.com/mas3/).

In order to identify groups of genes with similar changes in methylation in perirenal adipose tissue from offspring in the F1-DN and F1-CN groups, we defined the biological processes of the identified genes using a molecular annotation system. Genes were ranked by the level of significance (*p* values) according to the difference in methylation (Figure 6, Tables S1 and S2 in Supplementary Material available online at http://dx.doi.org/10.1155/2016/4952801). It is noteworthy that some genes associated with adipocytokine signaling pathway were significantly hypomethylated, including* Camkk2*,* Pck1*,* Rxrb*,* Adipoq*,* Tnf*,* Prkag3*,* Nfkbib*, and* Mapk10*. Moreover, the methylation level of genes related to type II diabetes mellitus was also prominently decreased, including* Prkce*,* Prkcz*,* Adipoq*,* Tnf*,* Slc2a2*,* Prkcd*, and* Mapk10* (Figure S1). As a key gene associated with adipocytokine signaling pathway and diabetes, the* Tnf* promoter was hypomethylated in perirenal adipose tissue from F1-DN group compared to controls (*p* < 0.05).

### 3.6. Methylation Levels of* Tnf* Promoter Region in F1 and F2 Offspring

**Figure 7:**
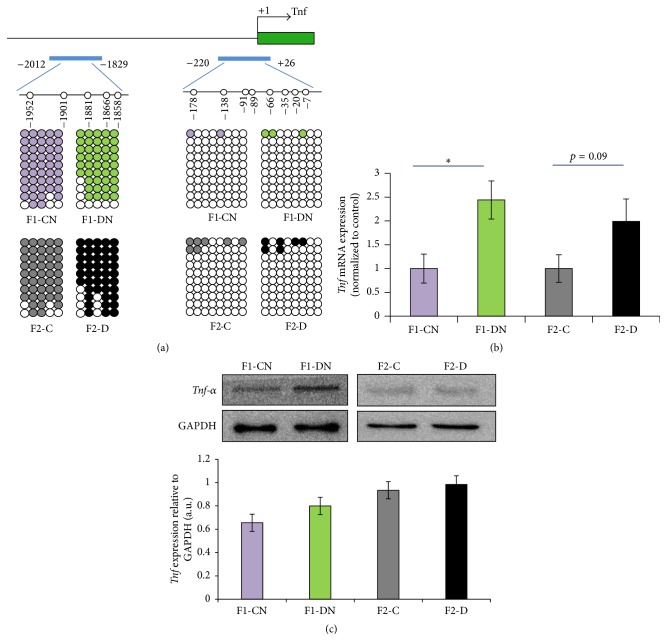
Methylation analysis and gene expression of* Tnf*. (a) Bisulfite sequencing was performed on the portion of the promoter regions encompassing −2012 to −1829 relative to the +1 transcription start site of the* Tnf* gene including 5 CpG sites and −220 to +26 including 8 CpG sites. Each line represents the average methylation level of the sequence of all the clones. CpG sites are shown as blank (unmethylated) or filled (methylated) circles (*N* = 3 per group). (b) The expression of* Tnf* was identified using real-time PCR (*N* = 8-9). (c) The protein expression of* Tnf* was identified using Western blotting (*N* = 5 per group). Results are presented as the means ± SEM. ^*∗*^ *p* < 0.05 versus F1-CN. *N* = 4–9.

MeDIP data showed that the portion of the* Tnf* promoter region from −2914 to −1717 was hypomethylated. Bisulfite sequencing was used to validate results of MeDIP for* Tnf*. Genomic DNA was extracted from perirenal adipose tissue obtained from F1 and F2 female offspring. Bisulfite sequencing was performed on a portion of the promoter region encompassing −2012 to −1829 relative to the +1 TSS of the* Tnf* gene, including 5 CpG sites (−1952, −1901, −1881, −1866, and −1858). We found a significant decrease in methylation in the −1952 site in the F1-DN group compared to the F1-CN group (Figure 7(a)). Interestingly, the hypomethylation level at the −1952 site was similar in the F2-D and the F1-DN groups (Figure 7(a)).

We further carried out bisulfite sequencing on another portion of the* Tnf* promoter region encompassing −220 to +26 relative to the +1 TSS, which included 8 CpG sites (−178, −138, −91, −89, −66, −35, −20, and −7). The methylation level did not significantly differ between the F1-DN and F1-CN groups, and similar results were obtained for the F2 generation (Figure 7(a)).

### 3.7. *Tnf* Methylation May Control the Expression of* Tnf*

In order to investigate the physiological importance of methylation in gene and protein expression, we measured the* Tnf* gene and protein expression levels using real-time PCR and Western blot analysis, respectively. The* Tnf* gene expression was significantly higher in the F1-DN group than in the F1-CN group (*p* < 0.05), and the* Tnf* gene expression showed an increasing trend in the F2-D group compared to the F2-C group, but this difference was not statistically significant (*p* = 0.09; Figure 7(b)). The protein expression did not significantly differ in the F1-DN and F2-D groups compared to the corresponding control groups (Figure 7(c)).

Hypomethylation of the* Tnf* promoter region at −1952 site was negatively correlated with the increased gene expression in diabetic offspring (*r* = −0.36). The transcription factors* Tfap2a*,* Tfap2c*,* Ebf1*,* Egr1*,* Rxra*,* c-Rel*,* RelA*, and* Nfic* predicted by the JASPAR database may be involved in the regulation of* Tnf* expression by binding to the specific CpG site in a methylation-sensitive manner.

### 3.8. Expression of Inflammatory Cytokines in Perirenal Adipose Tissue

**Figure 8:**
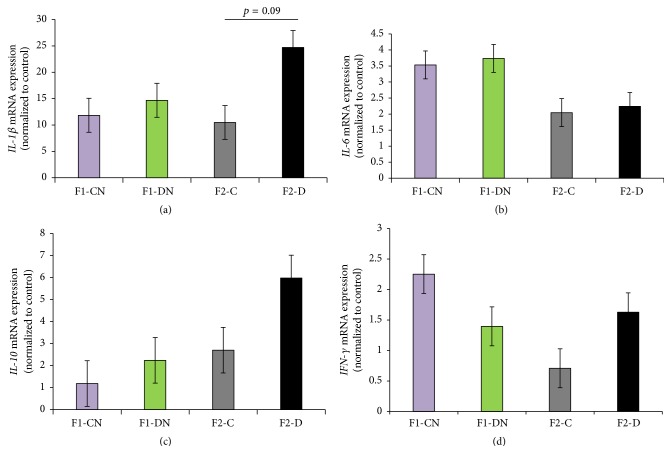
Inflammatory cytokines in perirenal adipose tissue. (a)* IL-1β* expression was identified using real-time PCR. (b)* IL-6* expression was identified using real-time PCR. (c)* IL-10* expression was identified using real-time PCR. (d)* IFN-γ* expression was identified using real-time PCR. Results are presented as the means ± SEM. *N* = 4-5.

Expression of inflammatory cytokines in perirenal adipose tissue was detected, and the expression levels of* IL-1β*,* IL-6*,* IL-10*, and* IFN-γ* did not significantly differ between the F1-DN and F2-D groups compared to controls (Figure 8).

## 4. Discussion

A GDM rodent model can be established using streptozotocin, which is directly toxic to maternal pancreatic *β*-cells. A previous study found that small fetuses were delivered following severe intrauterine hyperglycemia induced by a high dose of streptozotocin [8]. Nevertheless, GDM women with moderate hyperglycemia and large babies are more common in the clinical setting. Previous studies have found that administering a low dose of streptozotocin for five consecutive days can also induce moderate intrauterine hyperglycemia [8]. We established a GDM rat model with a single intraperitoneal injection of 25 mg/kg streptozotocin. The resultant moderate glucose level and the large fetus were used to mimic human macrosomia in offspring from mothers with GDM.

The birth weight of F1 offspring was significantly higher in offspring of the GDM group than in the control group; furthermore, the body weight and fat mass were increased in the offspring during adulthood. Although the F1 offspring of mothers in the GDM group had normal blood glucose levels at birth, impaired glucose tolerance and abnormal insulin and HDL levels were displayed in adulthood. The pancreatic islets were smaller in size and disordered and the insulin secretion was affected in F1 offspring of mothers with GDM, indicating that intrauterine hyperglycemia may lead to abnormal structure of fetal islets. The impaired *β*-cells may be related to the development of IGT.

The birth weights did not significantly differ between F2 offspring of mothers with GDM and controls. Nevertheless, the F2 offspring showed higher body weight and fat mass in adulthood. Furthermore, F2 offspring displayed hyperglycemia at birth and increased insulin secretion from the pancreatic islets in adulthood.

Together, these data contribute to the idea that early-life exposure to hyperglycemia in utero can influence glucose metabolism and induce insulin resistance in offspring up to the F2 generation. In addition, transgenerational offspring will be prone to obesity and hyperlipidemia in adulthood. It is indicated that a certain developmental pattern is programmed in utero. Ding et al. [9] demonstrated that intrauterine hyperglycemia induced IGT and abnormal insulin levels in both F1 and F2 offspring, which are partly due to the deficient islet ultrastructure. Future studies should aim to investigate the potential mechanisms by which environmental factors can influence the expression of genes involved in insulin resistance.

Previous studies have showed that a low-grade inflammation characterized by increased circulating levels of proinflammatory cytokines in adipose tissue, such as* IL-6* and* Tnf*, and chemokines is a common feature of obesity and diabetes [10]. The overexpression of* Tnf* is closely related to insulin resistance, reduced lipoprotein lipase activity, and increased lipase activity. Moreover,* Tnf* is an adipokine involved in systemic inflammation, and inflammatory factors secreted from adipocytes may interfere with the insulin signaling pathway and impair the action of insulin and glucose transport [11–14]. In our study,* Tnf* expression was increased in the F1 generation born from mothers with GDM and relatively increased in F2 offspring. Additionally, chronic inflammation was found in adipose tissue, and the levels of* IL-1β* and* IL-10* tended to increase in both F1 and F2 offspring obtained from F0 mothers with GDM. Transgenerational transmission of inflammatory state may be presented between F1 and F2 offspring. Consistent with our study, Ding et al. [15] showed that a feed-forward cycle exists in female mice after continuous high-fat diet stress, as demonstrated by increased adiposity and progressive inflammation in adipose tissue across generations.

Epigenetic mechanisms have been cited as a possible link between environmental and nutritional factors and the regulation of gene expression. DNA methylation is a major epigenetic modification. Evidence suggests that gene expression can be regulated by DNA methylation in diabetic patients, and the differential methylation may be responsible for the progression of type 2 diabetes [16]. Environmental events and nutritional conditions during early pregnancy may induce permanent DNA methylation changes in the fetus in utero. These studies emphasized the effect of early intrauterine exposure on epigenetic changes, which may be “memorized” and last throughout the lifetime of the offspring. Additionally, these epigenetic changes could also be transmitted to the F2 generation and show transgenerational effects [17, 18]. Nevertheless, most studies in this area have only focused on restricted intrauterine nutrition and reported the epigenetic modifications in intrauterine growth restriction (IUGR) models [19–21]. Our study is the first to provide further evidence of intrauterine exposure to excess nutrition.

We identified specific changes in DNA methylation induced by intrauterine hyperglycemia using MeDIP array. We identified a total of 923 and 2150 promoters in the visceral adipose tissue of F1 offspring that had been significantly hypermethylated and hypomethylated, respectively. It is noteworthy that the* Tnf* promoter was hypomethylated in diabetic offspring compared to the controls. Further bisulfite sequencing of the* Tnf* promoter encompassing −220 to +26 revealed that, overall, hypomethylation did not broadly alter the chromatin in diabetic offspring; rather, it was confined to the specific cytosine.

The* Tnf* promoter sequence contains CpG dinucleotides within or next to transcription factor-binding sites. The CpG site at site −1952 of the* Tnf* promoter was distinctly hypomethylated both in F1 and F2 offspring of diabetic mothers and the 1952-bp 5′-flanking region of the promoter may be a* c-Rel*-,* EBF1*-,* NFIC*-,* RelA*-, and* NF-κB*-binding site. Therefore, DNA methylation is proposed to participate in regulating the expression of the* Tnf* gene by interfering with transcription binding sites, playing a role in the progression of insulin resistance. Consistent with our findings, a study by Lou et al. [22] showed that the activation and upregulation of proinflammatory cytokines such as* IL-1β*,* IL-6*,* TNF-α*, and* IFN-γ* mainly occurred in the arterial intima of diabetic rats, and reduced levels of DNA methylation at the specific cytosine-phosphate-guanosine sites of* IL-1β*,* IL-6*,* TNF-α*, and* IFN-γ* may be one of the mechanisms to regulate the expression of these genes. Our results emphasized the effect of early intrauterine exposure on insulin sensitivity via epigenetic changes, which may persist throughout the lifetime as well as get transmitted to the next generation.

Another factor related to insulin resistance is lifestyle, and previous studies have reported that accumulation of intracellular lipid metabolites from incomplete lipid oxidation could inhibit signal transduction to glucose transport [23–25], suggesting the involvement of a high-fat diet in the development of insulin resistance. In the current study, offspring were given high-fat diets, and we found that fat overload could exaggerate the programmed glucose intolerance in offspring exposed to intrauterine hyperglycemia. The underlying mechanism for this phenomenon is not yet fully understood. Barrès et al. reported that elevated free fatty acids might induce insulin resistance through epigenetic mechanisms [16].

## 5. Conclusions

Overall, our results revealed that intrauterine hyperglycemia can influence the DNA methylation level in the promoter regions of inflammatory factors involved in insulin resistance, such as* TNF-α*, and the DNA methylation is intergenerational and inherited.

## Supplementary Material

Supplementary Table 1. Pathways of changes in hypermethylation in F1 offspring exposed to intrauterine hyperglycemia. Genes with changes in hypermethylation in perirenal adipose tissue from offpring in the F1-DN and F1-CN groups are identified using a molecular annotation system.
Supplementary Table 2. Pathways of changes in hypomethylation in F1 offspring exposed to intrauterine hyperglycemia.
Supplementary Figure 1. Differences in methylation status of genes related to the adipocytokine signaling pathway and type II diabetes. (A) Differences in hypermethylation in genes related to type II diabetes. (B) Differences in hypomethylation in genes related to the adipocytokine signaling pathway and type II diabetes. Log ratios showed the methylation difference.

## Figures and Tables

**Table 1:** Body weight (grams) of F1 and F2 females.

	*N*	Birth	3 weeks	7 weeks	11 weeks	15 weeks	19 weeks	23 weeks	28 weeks
F1-CN	8	6.26 ± 0.13	37.51 ± 1.25	171.88 ± 4.73	242.00 ± 7.70	259.38 ± 7.52	284.25 ± 10.70	301.13 ± 11.02	309.50 ± 12.84
F1-DN	9	6.90 ± 0.14^a^	41.93 ± 4.08	179.11 ± 7.86	255.56 ± 9.29	284.33 ± 8.57^a^	305.44 ± 8.92	318.22 ± 10.11	325.44 ± 10.91
F1-CF	8	6.37 ± 0.08	38.01 ± 1.68	169.38 ± 4.38	248.63 ± 6.70	281.50 ± 8.20^a^	311.50 ± 11.34	328.38 ± 14.23	332.57 ± 15.16
F1-DF	8	7.04 ± 0.09^b^	42.56 ± 1.91	179.50 ± 4.32	259.50 ± 7.08	292.50 ± 9.43	306.86 ± 10.07	317.43 ± 14.78	318.86 ± 14.85
F2-C	6	7.2 ± 0.4	57.62 ± 1.49	187 ± 3.96	248.66 ± 6.11	274.17 ± 3.7	286.5 ± 7.53	288.66 ± 9.61	300.17 ± 6.63
F2-D	6	7.1 ± 0.8	56.80 ± 1.94	198.83 ± 6.69	256.66 ± 9.59	284 ± 9.08	299.17 ± 6.62	310.83 ± 5.64^*∗*^	322.33 ± 6.65^*∗*^

Data are means ± SEM. Significance was determined by ANOVA. ^a^ *p* < 0.05 versus F1-CN, ^b^ *p* < 0.05 versus F1-CF, and ^*∗*^ *p* < 0.05 versus F2-C.

**Table 2:** Body composition (grams) of F1 and F2 females at 28 weeks.

	*N*	Mesenteric adipose tissue weight (g)	Perirenal adipose tissue weight (g)	Ovarian adipose tissue weight (g)	Visceral adipose tissue weight (g)	Pancreas weight (g)	Liver weight (g)	Heart weight (g)	Kidney weight (g)
F1-CN	8	4.34 ± 0.79	3.79 ± 0.89	7.69 ± 1.42	15.82 ± 3.03	1.97 ± 0.06	7.68 ± 0.21	0.92 ± 0.04	1.98 ± 0.06
F1-DN	7	3.78 ± 0.44	4.21 ± 0.42	9.57 ± 0.96	17.56 ± 1.72	1.96 ± 0.08	7.67 ± 0.46	0.89 ± 0.04	1.96 ± 0.08
F1-CF	7	5.34 ± 1.18	6.93 ± 1.96	11.88 ± 2.10	24.16 ± 4.99	1.90 ± 0.10	7.13 ± 0.47	0.90 ± 0.05	1.90 ± 0.10
F1-DF	4	3.9 ± 1.01	4.28 ± 0.92	8.87 ± 1.92	17.05 ± 3.81	1.98 ± 0.06	8.49 ± 0.64	0.77 ± 0.03	1.98 ± 0.06
F2-C	8	3.87 ± 0.43	3.56 ± 0.30	9.95 ± 0.44	17.37 ± 0.75	0.91 ± 0.09	9.31 ± 0.33	0.91 ± 0.03	2.38 ± 0.05
F2-D	8	4.18 ± 0.63	4.49 ± 0.27^*∗*^	8.88 ± 0.69	17.89 ± 1.46	0.89 ± 0.07	8.68 ± 0.44	0.89 ± 0.02	2.11 ± 0.34

Data are means ± SEM. Significance was determined by ANOVA. ^*∗*^ *p* < 0.05 versus F2-C.

## Abbreviations

GDM: Gestational diabetes mellitus
MeDIP: Methylated DNA immunoprecipitation
STZ: Streptozotocin
GTT: Glucose tolerance test
IGT: Impaired glucose tolerance
IUGR: Intrauterine growth restriction
OGTT: Oral glucose tolerance test
IP: Immunoprecipitated
TNF: Tumor necrosis factor
IL-1*β*: Interleukin-1*β*
IL-6: Interleukin-6
IL-10: Interleukin-10
IFN-*γ*: Interferon-*γ*.

## References

[1] Zhu W. W., Yang H. X., Wei Y. M. (2013). Evaluation of the value of fasting plasma glucose in the first prenatal visit to diagnose gestational diabetes mellitus in China. *Diabetes Care*.

[2] Franks P. W., Looker H. C., Kobes S. (2006). Gestational glucose tolerance and risk of type 2 diabetes in young Pima Indian offspring. *Diabetes*.

[3] Manderson J. G., Mullan B., Patterson C. C., Hadden D. R., Traub A. I., McCance D. (2002). Cardiovascular and metabolic abnormalities in the offspring of diabetic pregnancy. *Diabetologia*.

[4] Barker D. J. P. (1999). The fetal origins of type 2 diabetes mellitus. *Annals of Internal Medicine*.

[5] Izaola O., De Luis D., Sajoux I., Domingo J. C., Vidal M. (2015). Inflammation and obesity (lipoinflammation). *Nutrición Hospitalaria*.

[6] Buras E. D., Yang L., Saha P. (2015). Proinsulin-producing, hyperglycemia-induced adipose tissue macrophages underlie insulin resistance in high fat-fed diabetic mice. *The FASEB Journal*.

[7] Yan J., Li X., Su R., Zhang K., Yang H. (2014). Long-term effects of maternal diabetes on blood pressure and renal function in rat male offspring. *PLoS ONE*.

[8] Van Assche F. A., Holemans K., Aerts L. (2001). Long-term consequences for offspring of diabetes during pregnancy. *British Medical Bulletin*.

[9] Ding G.-L., Wang F.-F., Shu J. (2012). Transgenerational glucose intolerance with Igf2/H19 epigenetic alterations in mouse islet induced by intrauterine hyperglycemia. *Diabetes*.

[10] Kayser B. D., Toledo-Corral C. M., Alderete T. L., Weigensberg M. J., Goran M. I. (2015). Temporal relationships between adipocytokines and diabetes risk in Hispanic adolescents with obesity. *Obesity*.

[11] Hotamisligil G. S., Peraldi P., Budavari A., Ellis R., White M. F., Spiegelman B. M. (1996). IRS-1-mediated inhibition of insulin receptor tyrosine kinase activity in TNF-*α*- and obesity-induced insulin resistance. *Science*.

[12] Uysal K. T., Wiesbrock S. M., Marino M. W., Hotamisligil G. S. (1997). Protection from obesity-induced insulin resistance in mice lacking TNF-alpha function. *Nature*.

[13] Yuan M., Konstantopoulos N., Lee J. (2001). Reversal of obesity- and diet-induced insulin resistance with salicylates or targeted disruption of *Ikkβ*. *Science*.

[14] Vallerie S. N., Furuhashi M., Fucho R., Hotamisligil G. S. (2008). A predominant role for parenchymal c-Jun amino terminal kinase (JNK) in the regulation of systemic insulin sensitivity. *PLoS ONE*.

[15] Ding Y., Li J., Liu S. (2014). DNA hypomethylation of inflammation-associated genes in adipose tissue of female mice after multigenerational high fat diet feeding. *International Journal of Obesity*.

[16] Barrès R., Osler M. E., Yan J. (2009). Non-CpG methylation of the PGC-1*α* promoter through DNMT3B controls mitochondrial density. *Cell Metabolism*.

[17] Painter R. C., Osmond C., Gluckman P., Hanson M., Phillips D. I. W., Roseboom T. J. (2008). Transgenerational effects of prenatal exposure to the Dutch famine on neonatal adiposity and health in later life. *BJOG: An International Journal of Obstetrics & Gynaecology*.

[18] Burdge G. C., Slater-Jefferies J., Torrens C., Phillips E. S., Hanson M. A., Lillycrop K. A. (2007). Dietary protein restriction of pregnant rats in the F0 generation induces altered methylation of hepatic gene promoters in the adult male offspring in the F1 and F2 generations. *British Journal of Nutrition*.

[19] Park J. H., Stoffers D. A., Nicholls R. D., Simmons R. A. (2008). Development of type 2 diabetes following intrauterine growth retardation in rats is associated with progressive epigenetic silencing of Pdx1. *The Journal of Clinical Investigation*.

[20] Fu Q., McKnight R. A., Callaway C. W., Yu X., Lane R. H., Majnik A. V. (2015). Intrauterine growth restriction disrupts developmental epigenetics around distal growth hormone response elements on the rat hepatic IGF-1 gene. *The FASEB Journal*.

[21] Goodspeed D., Seferovic M. D., Holland W. (2015). Essential nutrient supplementation prevents heritable metabolic disease in multigenerational intrauterine growth-restricted rats. *The FASEB Journal*.

[22] Lou X. D., Wang H. D., Xia S. J., Skog S., Sun J. (2014). Effects of resveratrol on the expression and DNA methylation of cytokine genes in diabetic rat aortas. *Archivum Immunologiae et Therapia Experimentalis*.

[23] Petersen K. F., Dufour S., Befroy D., Garcia R., Shulman G. I. (2004). Impaired mitochondrial activity in the insulin-resistant offspring of patients with type 2 diabetes. *The New England Journal of Medicine*.

[24] Ritov V. B., Menshikova E. V., He J., Ferrell R. E., Goodpaster B. H., Kelley D. E. (2005). Deficiency of subsarcolemmal mitochondria in obesity and type 2 diabetes. *Diabetes*.

[25] Kim J.-Y., Hickner R. C., Cortright R. L., Dohm G. L., Houmard J. A. (2000). Lipid oxidation is reduced in obese human skeletal muscle. *American Journal of Physiology—Endocrinology and Metabolism*.

