# Supplementary material for: Transgenerational Glucose Intolerance of Tumor Necrosis Factor with Epigenetic Alteration in Rat Perirenal Adipose Tissue Induced by Intrauterine Hyperglycemia
Source: J Diabetes Res. 2016 Jan 5;2016:4952801. doi: 10.1155/2016/4952801 (PMC4735892; doi:10.1155/2016/4952801)

Supplementary Table 1. Pathways of changes in hypermethylation in F1 offspring exposed to intrauterine hyperglycemia

Pathway	Enrichment Score (-log10[P value])	p-value	genes
Glycosaminoglycan degradation	2	0.006197	Hyal2;Hyal1
Fatty acid elongation in mitochondria	2	0.006197	Hadha;Echs1
Glycosphingolipid biosynthesis - globoseries	2	0.006197	St3gal2;B3galt5
Arachidonic acid metabolism	4	0.005752	Ptgds;Pla2g6;Ptgds2;Ptges
Benzoate degradation via CoA ligation	2	0.004998	Hadha;Echs1
Basal cell carcinoma	4	0.003407	Fzd5;Fzd9;Wnt5b;Gli2
Hedgehog signaling pathway	4	0.003407	Bmp8a;Csnk1d;Wnt5b;Gli2
Leukocyte transendothelial migration	6	0.002983	Plcg2;Cldn3;Thy1;Vav2;Rhoh;Cldn22
Caprolactam degradation	2	0.002963	Hadha;Echs1
Type II diabetes mellitus	4	0.002729	Cacna1e;Prkcz;Tnf;Abcc8
Hematopoietic cell lineage	5	0.002344	Il1a;Il6ra;Tnf;Il11;Cd7
Lysine degradation	4	0.001815	MGC109340;Ogdhl;Hadha;Echs1
Long-term depression	5	0.001528	Npr1;Grm5;Pla2g6;Nos2;Itpr2
Primary immunodeficiency	4	8.31E-04	Ikbkg;Zap70;Rfxank;Tap2
ABC transporters - General	4	7.43E-04	Abca1;Tap2;Abcc8;Abcb6
N-Glycan degradation	3	4.64E-04	Aga;RGD1310868;Neu4
TGF-beta signaling pathway	6	3.79E-04	Crebbp;Smad5;Tgfb1;Bmp8a;Tnf;Rps6kb2
Proteasome	5	3.48E-04	Psmd7;Psmb7;Psmb11;Psmb10;Psmb8
Glycerophospholipid metabolism	5	3.16E-04	Ptdss1;Dgkz;Lcat;Pla2g6;Chkb
Biosynthesis of unsaturated fatty acids	4	2.59E-04	Elovl2;Hadha;Elovl5;Gpsn2
PPAR signaling pathway	6	2.27E-04	Angptl4;Slc27a6;Fabp6;Pparg;Cpt1b;Slc27a1

Tryptophan metabolism	5	2.11E-04	Aanat;Ogdhl;Hadha;Echs1;Mettl6
Complement and coagulation cascades	6	1.79E-04	F5;C8g;Masp1;F10;Serpinf2;C4b
Tight junction	8	1.68E-04	Llgl1;Pard6b;Myh7b;Cldn3;Prkcz;Src;Rras;Cldn22
Gap junction	7	1.24E-04	Csnk1d;Npr1;Tubb3;Grm5;Src;Itpr2;Tuba3a
Androgen and estrogen metabolism	5	9.28E-05	Hsd17b1;Sult2b1;Ugt1a2;Ugt1a1;Mettl6
Cytokine-cytokine receptor interaction	13	8.35E-06	Il1a;LOC500598;Il6ra;Cx3cr1;Tgfb1;Tnfsf13;Tnfrsf1a;Bmp8a;Cd27;Tnf;Lta;Il11;Ccl11
MAPK signaling pathway	16	1.48E-07	Il1a;Cacna1e;Ikbkg;Fgfr3;Tgfb1;Tnfrsf1a;Dusp1;Tnf;Pla2g6;Map4k3;Rras;Flnb;Cacna1i; Cacnb2;Map3k4;Srf
Calcium signaling pathway	19	2.58E-12	Cacna1e;Plcg2;Tbxa2r;Ptger1;Adrb2;P2rx7;Ntsr1;Ryr2;Adra1d;Htr5a;Tnnc2;Drd5;Grm5; Nos2;Adora2a;Itpr2; Chrm1;Gna15;Cacna1i
Neuroactive ligand-receptor interaction	26	7.87E-15	Grin2b;Tbxa2r;Prlh;Ptger1;Adrb2;P2rx7;Pth2r;Mc3r;Ntsr1;Tspo;Galr2;Oxt;Oprl1;Adra1d; Hrh4;Htr5a;Drd5; Aplnr;Grm5;Avp;Adora2a;Crhr2;Chrm1;Grp;Mas1;Ucn2

Supplementary Table 2. Pathways of changes in hypomethylation in F1 offspring exposed to intrauterine hyperglycemia

Pathway	Enrichment Score (-log10[P value])	p-value	gene
Adipocytokine signaling pathway	8	3.70E-04	Camkk2;Pck1;Rxrb;Adipoq;Tnf;Prkag3;Nfkbib;Mapk10
Glycerophospholipid metabolism	7	3.39E-04	Dgkb;Pemt;Cdipt;Agpat1;Lcat;Phospho1;Ppap2c
Glycosphingolipid biosynthesis - lactoseries	5	3.28E-04	B3gnt4;St3gal6;Fut7;Fut1;B4galt2
Type II diabetes mellitus	7	2.98E-04	Prkce;Prkcz;Adipoq;Tnf;Slc2a2;Prkcd;Mapk10
Insulin signaling pathway	12	2.86E-04	Pck1;Gys2;Ppp1r3b;Bad;Shc2;Inpp5d;Prkcz;Rps6kb2;Shc1;Prkar2a;Prkag3;Mapk10
TGF-beta signaling pathway	9	2.65E-04	Rbl2;Tgfb2;Ltbp1;Rps6kb2;Tnf;Tgfb1;Smad1;Id4;Bmp8a
GnRH signaling pathway	10	1.84E-04	Mmp14;Adcy4;Mmp2;Gnas;Map2k6;Mapk12;Prkcb;Prkcd;Itpr3;Mapk10
Citrate cycle (TCA cycle)	6	1.34E-04	Idh3a;Pck1;Idh2;Pdha1;Idh3g;Sdhb
VEGF signaling pathway	9	1.28E-04	Bad;Ppp3r2;Shc2;Mapk12;Mapkapk2;Nfatc4;Prkcb;Rac2;Ptk2
Leukocyte transendothelial migration	12	7.81E-05	Icam1;Mmp2;Mapk12;Ocln;Prkcb;Itgb2;Cldn9;Actn1;Rap1a;Rac2;Ptk2;Pecam1
Taste transduction	7	7.69E-05	Adcy4;Tas2r134;Gnas;Scnn1a;Itpr3;Tas2r126;Kcnb1
Hedgehog signaling pathway	8	6.52E-05	Csnk1g3;Csnk1g2;Sufu;Gli2;Wnt8a;Bmp8a;Wnt7b;Btrc
Natural killer cell mediated cytotoxicity	14	3.49E-05	Icam1;Slc39a7;Cd244;Ppp3r2;Shc2;Nfatc4;Prkcb;Itgb2;Shc1;Tnf;Tnfrsf10b;Rac2;Ifngr2;Ifna4
Purine metabolism	14	2.75E-05	Hprt1;Paics;Polr2h;Polr2hl;Nme3;Nt5e;Adcy4;Pde9a;Polr2i;Polr2c;Pde2a;Polr1c;Pde8b;Xdh
Cell adhesion molecules (CAMs)	15	1.57E-05	Cdh15;Icam1;Mag;Ctla4;Pvrl3;Slc39a7;Madcam1;LOC288521;Cntn1;L1cam;Ocln;Itgb2;Cldn9;Cd8a;Pecam1
Toll-like receptor signaling pathway	12	8.35E-06	Map3k7;Tlr5;Cxcl9;Tlr2;Cd14;Map2k6;Mapk12;Tnf;Irak4;Myd88;Mapk10;Ifna4
Hematopoietic cell lineage	11	6.43E-06	Tpo;Epo;Fcer2;Cd14;Anpep;Gp1bb;Csf3;Tnf;Cd8a;Cd37;Kit
PPAR signaling pathway	11	3.25E-06	Pck1;Rxrb;Acox2;Olr1;Cyp7a1;Ucp1;Adipoq;Acaa1;RGD1562373;Fads2;Aqp7

Wnt signaling pathway	16	1.44E-06	Map3k7;Ctbp1;Tbl1xr1;Apc2;Ppp3r2;Nfatc4;Prkcb;Wnt8a;Axin2;Ppp2r5b;Rac2;Prickle1; Wnt7b;Csnk2b;Mapk10;Btrc
Focal adhesion	19	1.03E-06	Flt4;Bcl2;Pdgfa;Egf;Bad;Mylk2;Shc2;Itga7;Itga11;Prkcb;Shc1;Ibsp;Actn1;Rap1a;Rac2; Itga10;Ptk2;Mapk10;Lamc3
Jak-STAT signaling pathway	17	2.85E-07	Tpo;Epo;Prlr;Il21;Csf3;Spred2;Osm;Il9;Cntfr;Clcf1;Stat5a;Prl;Il24;Ifngr2;Irf9;Il10rb;Ifna4
Tight junction	17	6.77E-08	Prkce;Prkch;Myh7b;LOC304000;Rab3b;Myh6;Tjp2;Myh3;Prkcz;Ocln;Prkcb; Rab13;Cttn;Cldn9;Actn1;Prkcd;Csnk2b
Regulation of actin cytoskeleton	24	2.27E-09	Was;Bdkrb2;Pdgfa;Apc2;F2r;Egf;Chrm2;Cd14;Mylk2;Bdkrb1;Itga7;Itga11; Itgad;Fgf21;Arhgef6;Itgb2;Gsn;Ssh3;Actn1;Wasf2;Rac2;Fgf16;Itga10;Ptk2
Axon guidance	19	1.53E-09	Dpysl2;Efna5;Efnb3;Sema6c;Sema3f;Efna4;Ppp3r2;L1cam;Epha2;Nfatc4; Plxnb3;Ephb3;Cdk5;Epha3;Robo1;Epha1;Rac2;Sema4f;Ptk2
Complement and coagulation cascades	17	2.56E-12	C4b;Bdkrb2;F10;Serping1;F2r;Bdkrb1;Serpinc1;F3;Fgb;Cfi;F8;Kng1;C1qa;C1qb;C4bpa; Serpina5;C2
MAPK signaling pathway	32	1.29E-12	Map3k7;Tgfb2;Mapk6;Ecsit;Pdgfa;Cacng5;Cacna1i;Egf;Cd14;Map2k6;Rasa2;Ppp3r2;Mapk12; Mapkapk2;Rps6ka1;Cacnb4;Nfatc4;Stk3;Prkcb;Fgf21;Cacng8;Rasgrp3;Ptpn5;Tnf;Tgfb1;Rap1a; Cacng1;Rac2;Ptpn7;Fgf16;Ntf4;Mapk10
Calcium signaling pathway	29	5.83E-14	Adra1a;Grpr;Bdkrb2;Atp2b3;Tnnc1;Adcy4;Cacna1i;Vdac3;F2r;Gnas;Chrm2;Ptgfr; Avpr1b;Mylk2;Ppp3r2;Ptger1;Bdkrb1; Nos1;Slc8a2;Prkcb;Plce1;Lhcgr;Adrb1;Drd1a;Htr6;Ltb4r2;P2rx1;Trhr2;Itpr3
Cytokine-cytokine receptor interaction	37	1.23E-17	Tpo;Flt4;Epo;Cx3cl1;Relt;Tgfb2;Cxcl9;Prlr;Ccl26;Il8rb;Pdgfa;Il18r1;Egf;Ccl17;Tnfrsf25; Il21;Csf3;Osm; Il9;Ltbr;Cntfr;Clcf1;Tnf;Lta;Tgfb1;Ccl4;LOC301289; Bmp8a;Kit;Tnfrsf21;Prl;Tnfrsf10b;Il24;Ifngr2;Il10rb;Ccr6;Ifna4
Olfactory transduction	72	1.41E-20	Olr1401;Olr1768;Olr139;Olr1454;Olr62;Olr140;Olr107;Olr1592;Olr495;Olr288;Olr859;Olr1335; Olr144;Olr837;Olr462;Olr1092;Olr45;Olr78;Olr1456;Olr1702;Olr1765;Olr1644;Olr156;Olr1612; Olr1387;

			Olr840;Olr856;Olr1645;<br>Olr84;Olr1l;Olr303;Olr857;Olr1432;Olr425;Olr1686;Olr321;Olr875;Olr43;Olr69;Olr448;<br>Olr1091;Olr4;Olr836;Olr1744;Olr153;Olr154;Olr1090;Olr1344;Olr434;Olr1568;Olr1413;<br>Olr838;Olr113;Olr300;Olr307;Olr1309;Olr142;Olr1657;Olr1337;<br>Olr149;Olr483;Olr130;Olr1595;Olr1334;Olr1096;Olr1106;Olr1198;Olr1306;Olr148;<br>Olr1111;Olr168;Olr1093
Neuroactive ligand-receptor interaction	50	8.78E-25	Gpr83;Adra1a;Glp1r;Grpr;Galr1;Ppyr1;P2ry14;Prlr;Fshr;Bdkrb2;Ptgir;Nmur1;Grp;<br>F2r;Ltb4r;Chrm2;Ptgfr;Tac4;<br>Avpr1b;Grm2;Ptger1;Bdkrb1;Hcrt;Gpr156;Mtnr1a;Gpr35;Gabrg2;Lhcgr;Adrb1;<br>Ppy;Grik5;S1pr4;Uts2;Ucn2;Drd3;<br>Taar1;Drd1a;Glp2r;Ucn2;P2ry13;Kiss1r;Htr6;Ltb4r2;Hcrtr1;Sstr3;Prl;P2rx1;<br>Trhr2;Tac2;P2ry4;Cnr2

Supplementary Figure 1

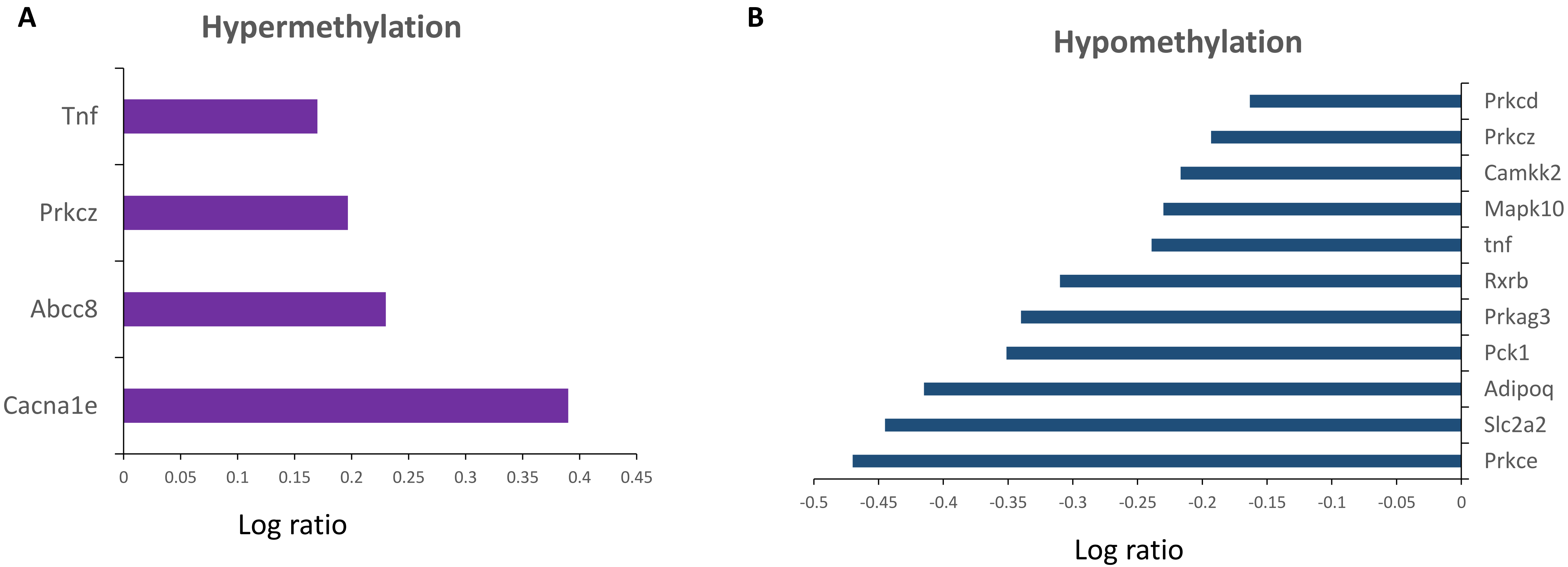

Supplement: Supplementary file 1 — Supplementary Table 1. Pathways of changes in hypermethylation in F1 offspring exposed to intrauterine hyperglycemia. Genes with changes in hypermethylation in perirenal adipose tissue from offpring in the F1-DN and F1-CN groups are identified using a molecular annotation system. Supplementary Table 2. Pathways of changes in hypomethylation in F1 offspring exposed to intrauterine hyperglycemia. Supplementary Figure 1. Differences in methylation status of genes related to the adipocytokine signaling pathway and type II diabetes. (A) Differences in hypermethylation in genes related to type II diabetes. (B) Differences in hypomethylation in genes related to the adipocytokine signaling pathway and type II diabetes. Log ratios showed the methylation difference. [file 4952801.f1.pdf]
